# The effects of sampling on the efficiency and accuracy of *k*−mer indexes: Theoretical and empirical comparisons using the human genome

**DOI:** 10.1371/journal.pone.0179046

**Published:** 2017-07-07

**Authors:** Meznah Almutairy, Eric Torng

**Affiliations:** Department of Computer Science and Engineering, Michigan State University, East Lansing, Michigan, United States of America; University of Helsinki, FINLAND

## Abstract

One of the most common ways to search a sequence database for sequences that are similar to a query sequence is to use a *k*-mer index such as BLAST. A big problem with *k*-mer indexes is the space required to store the lists of all occurrences of all *k*-mers in the database. One method for reducing the space needed, and also query time, is sampling where only some *k*-mer occurrences are stored. Most previous work uses *hard sampling*, in which enough *k*-mer occurrences are retained so that all similar sequences are guaranteed to be found. In contrast, we study *soft sampling*, which further reduces the number of stored *k*-mer occurrences at a cost of decreasing query accuracy. We focus on finding highly similar local alignments (HSLA) over nucleotide sequences, an operation that is fundamental to biological applications such as cDNA sequence mapping. For our comparison, we use the NCBI BLAST tool with the human genome and human ESTs. When identifying HSLAs, we find that soft sampling significantly reduces both index size and query time with relatively small losses in query accuracy. For the human genome and HSLAs of length at least 100 bp, soft sampling reduces index size 4-10 times more than hard sampling and processes queries 2.3-6.8 times faster, while still achieving retention rates of at least 96.6%. When we apply soft sampling to the problem of mapping ESTs against the genome, we map more than 98% of ESTs perfectly while reducing the index size by a factor of 4 and query time by 23.3%. These results demonstrate that soft sampling is a simple but effective strategy for performing efficient searches for HSLAs. We also provide a new model for sampling with BLAST that predicts empirical retention rates with reasonable accuracy by modeling two key problem factors.

## Introduction

We study the problem of trying to find the best sampling strategy to create simultaneously efficient and accurate *k*-mer indexes. These *k*-mer indexes have been widely used to accelerate the process of searching for all *highly similar local alignments* (HSLAs) between a query sequence and a database of sequences. This is a fundamental operation for a wide variety of biological applications including homologous search [1–4], detection of single nucleotide polymorphisms (SNP) [5–7], and mapping cDNA sequences against the corresponding genome [8–10]. We focus on finding HSLAs over nucleotide sequences where nucleotides are represented by A, C, G, and T. The HSLAs are commonly used in applications that compare sequences within the same species or closely related species, and we restrict our study’s database to the human genome.

One of the biggest problems with using *k*-mer indexes is that the size of the index is significantly larger than the underlying database. As biological databases/data sets rapidly increase in size, the size of the resulting *k*-mer indexes make using a *k*-mer index infeasible in many applications. Furthermore, query time increases rapidly as the database’s size and/or the number of queries increases. To ensure *k*-mer indexes remain viable, we must mitigate *k*-mer index size and query time.

One of the most effective and widely used ways of mitigating *k*-mer index size and query time is to perform sampling, in which we omit some *k*-mer occurrences from the index. In this paper, we study how best to sample a *k*-mer index to manage index size, query time, and accuracy where accuracy refers to finding all desired HSLAs. We evaluate a wide range of sampling rates that includes existing sampling practices as well as many new sampling rates. In particular, we study *soft sampling*, or sampling especially sparsely to further reduce index size and query time at the risk of missing some HSLAs. We show that using soft sampling, which has largely been ignored in previous studies, significantly reduces index size and computation times with very little loss in accuracy.

### Application 1: Highly similar local alignments

We study *k*-mer indexes in the context of two motivating applications. The first and primary application is finding HLSAs between a query sequence *q* and a database of sequences *DB*. The second application is to map ESTs to a genome, first finding HSLAs between the ESTs and the genome. We now formally define the first application, finding HSLAs.

We start by defining a local alignment *A*(*s*, *q*) between two sequences *s* and *q*. For simplicity, we denote *A*(*s*, *q*) as just *A*.

**Definition 1 (Local alignment)** *A local alignment* *A*(*s*, *q*) *between any two sequences* *s* *and* *q* *is a triple* (*x*, *y*, *m*) *where* *x* *is a contiguous subsequence of* *s*, *y* *is a contiguous subsequence of* *q*, *and* *m* *is an injective and monotonically increasing mapping from positions in* *x* *to positions in* *y*.

Within an alignment *A* = (*x*, *y*, *m*), some positions in *x* may map to no positions in *y* and vice versa. Let *map*(*A*) denote the number of positions in *x* that map to positions in *y*, and let *match*(*A*) denote the number of mapped positions that are identical. We then define the length of *A* to be |*A*| = |*x*| + |*y*| − *map*(*A*), and we define the match percentage to be *mp*(*A*) = *match*(*A*)/|*A*|. Finally, we define *E*(*A*) = |*A*| − *match*(*A*) to be the number of errors in alignment *A*.

**Fig 1:**
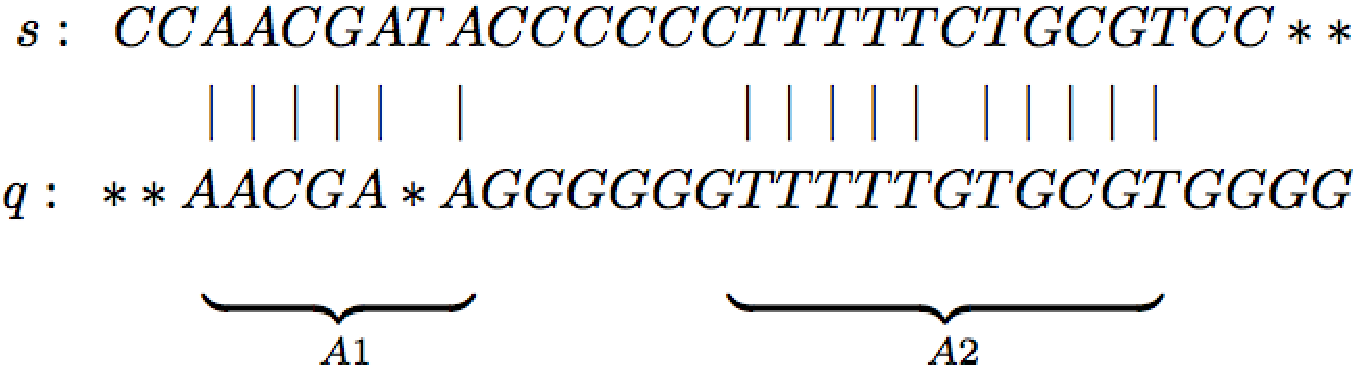
Example database sequence *s* and query sequence *q* and two local alignments *A*_1_ and *A*_2_, where (|) identifies to two mapped identical positions and (*) is an inserted gap position in one of the two sequences.

To illustrate these definitions, consider the example in Fig 1 with two local alignments *A*1 and *A*2. We have *map*(*A*1) = 6, *match*(*A*1) = 6, |*A*1| = 7, *mp*(*A*1) = 85.7%, and *E*(*A*) = 1 whereas *map*(*A*2) = 11, *match*(*A*2) = 10, |*A*2| = 11, *mp*(*A*2) = 91%, and *E*(*A*) = 1.

When searching for local alignments, our goal is to find all HSLAs that have a minimum length and match percentage. We formally define our targeted HSLAs, which we also refer to as true matches, as follows:

**Definition 2 (True match or HSLA)** *For a database of sequences* *DB*, *a query sequence* *q*, *an alignment length threshold* *l*, *and a match threshold* *t*, *we define* *HSLA*(*DB*, *q*, *l*, *t*) = {*A*(*s*, *q*) ∣ *s* ∈ *DB*,|*A*(*s*, *q*)| ≥ *l* *and* *mp*(*A*(*s*, *q*)) ≥ *t*}.

We use *HSLA*(*q*, *t*) when *DB* and *l* are clear from context. We also define short HSLAs to represent HSLAs that are barely in *HSLA*(*DB*, *q*, *l*, *t*) and are the hardest to find.

**Definition 3 (Short HSLA)** *For a database of sequences* *DB*, *a query sequence* *q*, *an alignment length threshold* *l*, *and a match threshold* *t*, *we define* *HSLA*_short_(*DB*, *q*, *l*, *t*) = {*A*(*s*, *q*) ∣ *s* ∈ *DB*, *l* ≤ |*A*(*s*, *q*)| ≤ (2 − *t*)*l* *and* *mp*(*A*(*s*, *q*)) ≥ *t*}.

For example, *HSLA*({*s*}, *q*, 6, 85%) = {*A*1, *A*2} whereas *HSLA*({*s*}, *q*, 11, 85%) = {*A*2}; *A*1 is omitted because it does not meet the length threshold of 11. Likewise, *HSLA*({*s*}, *q*, 6, 90%) = {*A*2}; *A*1 is dropped because it does not meet the match percentage threshold. Focusing on short HSLAs, *HSLA*_short_({*s*}, *q*, 6, 85%) = {*A*1}. *A*2 is dropped because it is too long. Note that *HSLA*({*s*}, *q*, 6, 90%) actually includes several alignments that overlap significantly with *A*1; we follow standard practice and only include the longest alignment with highest match percentage from any group of highly overlapping alignments in *HSLA*(*s*, *q*, *l*, *t*).

### Finding HSLAs using indexed BLAST

We now describe how indexed BLAST [4] is typically used to find HSLAs in *HSLA*(*DB*, *q*, *l*, *t*). Specifically, indexed BLAST uses a seed-and-extend search process where we have one *seed* phase and two *extension* phases. In the seed phase, for a given *k* value *k*′, indexed BLAST uses a *k*′-mer index to find shared *k*′-mers, where a shared *k*′-mer is a substring formed by *k*′ consecutive letters that appear in both a database sequence *s* ∈ *DB* and in the query *q*. More specifically, indexed BLAST identifies the locations or occurrences of these shared *k*′-mers. Once shared *k*′-mer occurrences are found, BLAST performs the first extension phase. In this phase, each occurrence is extended in both directions to find a maximal exact match (MEM), which is an exact match that cannot be extended in either direction without introducing mismatches. If a found MEM has length at least some threshold k* (defined below), BLAST performs the second extension phase where it tries to extend the MEM into an HSLA. BLAST’s extension process in this second phase is slightly more complex than the process from its first phase since BLAST must allow some mismatches and gaps in this second phase.

To illustrate this process, consider our example from Fig 1 and suppose we use BLAST to search for *HSLA*({*s*}, *q*, 11, 90%) with *k*′ = 4 and k* = 5. Suppose the seed phase returns the shared 4-mers AACG, TTTT, and TGCG. When BLAST performs the first extension phase, it would find the MEMs AACGA, TTTTT, and TGCGT. Since k* = 5, BLAST would then try to extend the three MEMs to HSLAs. The latter two would extend to *A*2 whereas the MEM AACGA cannot be extended into an HSLA.

We now describe BLAST’s first two phases in more detail starting with the seed phase. BLAST constructs a *k*′-mer index as follows. The *k*′-mer index saves a list of database *k*′-mers in a lookup table of all possible *k*′-mers, which is 4^*k*′^ entries. We refer to this lookup table as a dictionary. For each *k*′-mer in the dictionary, BLAST saves some of its occurrences in an inverted list (also known as an offset list). A *k*′-mer occurrence is an ordered pair (*s*, *i*) where *s* is the string containing this occurrence and *i* is the position of the last character in this occurrence.

BLAST then finds shared *k*′-mers as follows. BLAST extracts all *k*′-mers from query sequence *q*. BLAST then searches for each extracted *k*′-mer in the dictionary. If the extracted *k*′-mer is in the dictionary, it represents a shared *k*′-mer for *q* and some *s* ∈ *DB*. BLAST uses that *k*′-mer’s inverted list to find occurrences of that *k*′-mer in *DB*.

A key choice is what value of *k*′ should be used. Typically, *k*′ is chosen to be at most 16 so that the list of all possible *k*′-mers, which has 4^*k*′^ entries, can be stored as an array in RAM. Since we use BLAST to perform our experiments, we use BLAST’s default value of *k*′ = 12.

We next describe the first extension phase where BLAST searches for MEM_k_*s, which are MEMs of length at least k*. The extension itself is straightforward since mismatches and gaps are not allowed. The key issue for this phase is what k* should be. We want k* to be as large as possible to reduce the number of false positives, which are MEM_k_*s that cannot be extended into HSLAs. It is well known how to compute k* given a target alignment length *L* and a maximum number of errors *E* [11–13]. Specifically, k* = ⌊*L*/(1 + *E*)⌋. The basic idea is that the worst case is when the errors are evenly spaced. The question then is what value of *L* and *E* should be used. The hardest HSLAs to find are the short HSLAs defined in Definition 3; basically those of length exactly *l* and match percentage *t*. Thus, we use *L* = *l* and *E* = ⌊(1 − *t*)*l*⌋, which leads to k* = *l*/(1 + ⌊(1 − *t*)*l*⌋).

### Hard versus Soft sampling

The fundamental issue with using *k*′-mer indexes to search for HSLAs is that the *k*′-mer index can be very large. Most systems including BLAST control dictionary size by limiting *k*′ to a small value such as 12. With this choice of *k*′, the problem is that there are too many *k*′-mer occurrences because the total number of *k*′-mer occurrences is roughly the total length of all the sequences in the database *DB*. The human genome is roughly 3 billion base pairs, so this would mean roughly 3 billion *k*′-mer occurrences.

For this reason, *k*′-mer indexes are typically sampled where we only save some *k*′-mer occurrences rather than all of them. We focus on *fixed sampling* where for a given sampling step *w* ≥ 1, a *k*-mer that occurs at every *w*th position is saved. We distinguish between two types of fixed sampling: *hard sampling* [4, 9] and *soft sampling* [8].

In **hard sampling**, we choose *w* ≤ k* − *k*′ + 1 so that we are guaranteed to find a *k*′-mer within every MEM_k_*. Thus, when we apply the first extension step, we will find the resulting MEM_k_*. Since we find all MEM_k_*s after the first extension step, we are guaranteed to find all HSLAs after the second extension step. Without loss of generality, for hard sampling, we assume *w* = k* − *k*′ + 1 since this maximizes the space savings with no loss in accuracy. We refer to this *w* value as *w*_0_ (*w*_0_ = k* − *k*′ + 1).

In **soft sampling**, we consider *w* > *w*_0_. Because we no longer are guaranteed to choose a *k*′-mer from every MEM_k_*, when we apply the first extension phase, we may miss some MEM_k_*s, which may lead to missing some HSLAs in the next extension phase. Thus, if we use soft sampling, we risk missing some HSLAs.

### Retention rates and false positives

Recall our goal is to find *HSLA*(*DB*, *q*, *l*, *t*). We denote the HSLAs and short HSLAs found by using indexed BLAST with parameter values *k*′ and *w* to be *HSLA*(*DB*, *q*, *l*, *t*, *k*′, *w*) and *HSLA*_short_(*DB*, *q*, *l*, *t*, *k*′, *w*), respectively. With hard sampling (*w* = *w*_0_), we know *HSLA*(*DB*, *q*, *l*, *t*, *k*′, *w*_0_) = *HSLA*(*DB*, *q*, *l*, *t*). With soft sampling (*w* > *w*_0_), *HSLA*(*DB*, *q*, *l*, *t*) − *HSLA*(*DB*, *q*, *l*, *t*, *k*′, *w*) ≠ ∅ is possible. We define the retention rate of HSLAs as a function of *w* as follows.

**Definition 4 (Retention Rate)** *For a* *k*′-*mer index with a sampling step* *w*, *the retention rate for* *HSLA* *is* *RR*(*w*, *w*_0_) = |*HSLA*(*DB*, *q*, *l*, *t*, *k*′, *w*)|/|*HSLA*(*DB*, *q*, *l*, *t*)|, *and the retention rate for* *HSLA*_short_ *is* *RR*_*short*_(*w*, *w*_0_) = |*HSLA*_short_(*DB*, *q*, *l*, *t*, *k*′, *w*)|/|*HSLA*_short_(*DB*, *q*, *l*, *t*)|.

We typically express these ratios as percentages. We will study how *RR*(*w*, *w*_0_) and *RR*_*short*_(*w*, *w*_0_) change as a function of *w*. Because short HSLAs are the hardest true matches to find, we expect *RR*(*w*, *w*_0_) > *RR*_*short*_(*w*, *w*_0_) in most cases.

We present a new analytical model to compute the expected retention rate of HSLAs in *HSLA*_short_(*DB*, *q*, *l*, *t*). The new model is an extension to Kent’s analytical model [8] where he essentially assumed *w* = *k*′ = k*. On the other hand, we propose a new model where we assume *k*′ < k* and *w* ≥ 1. We refer to the new model as the BLAST model since it accounts for typical parameters used in BLAST searches.

Searching with sampled *k*′-mer index produces two intermediate results: shared *k*′-mers and MEM_k_*s. The second extension process, extending MEM_k_*s into HSLAs, is more complex and costly than the first extension process since we are allowing some mismatches and gaps. We thus define MEM_k_*s that do not extend into HSLAs to be **false positives**. We will also study how the number of false positives changes as a function of *w*.

### Application 2: Using *k*-mer indexes in EST mapping

Our second motivating application, which builds upon the first, is mapping ESTs on a genome, a fundamental procedure in genome research. These mappings are used to discover the intron-exon structure of genes, SNPs, and cDNA insertions and deletions, to name just a few applications. Many different mapping tools are available, each with their own advantages [14]. We focus on hash table–based, seed-and-extend mappers such as mrFAST/mrsFAST [15, 16], SHRiMP [17], Hobbes [18], drFAST [19], and RazerS [20]. These mappers are typically fully sensitive mappers that “can detect reads missed by other tools” [14] but may be relatively slow.

**Fig 2:**
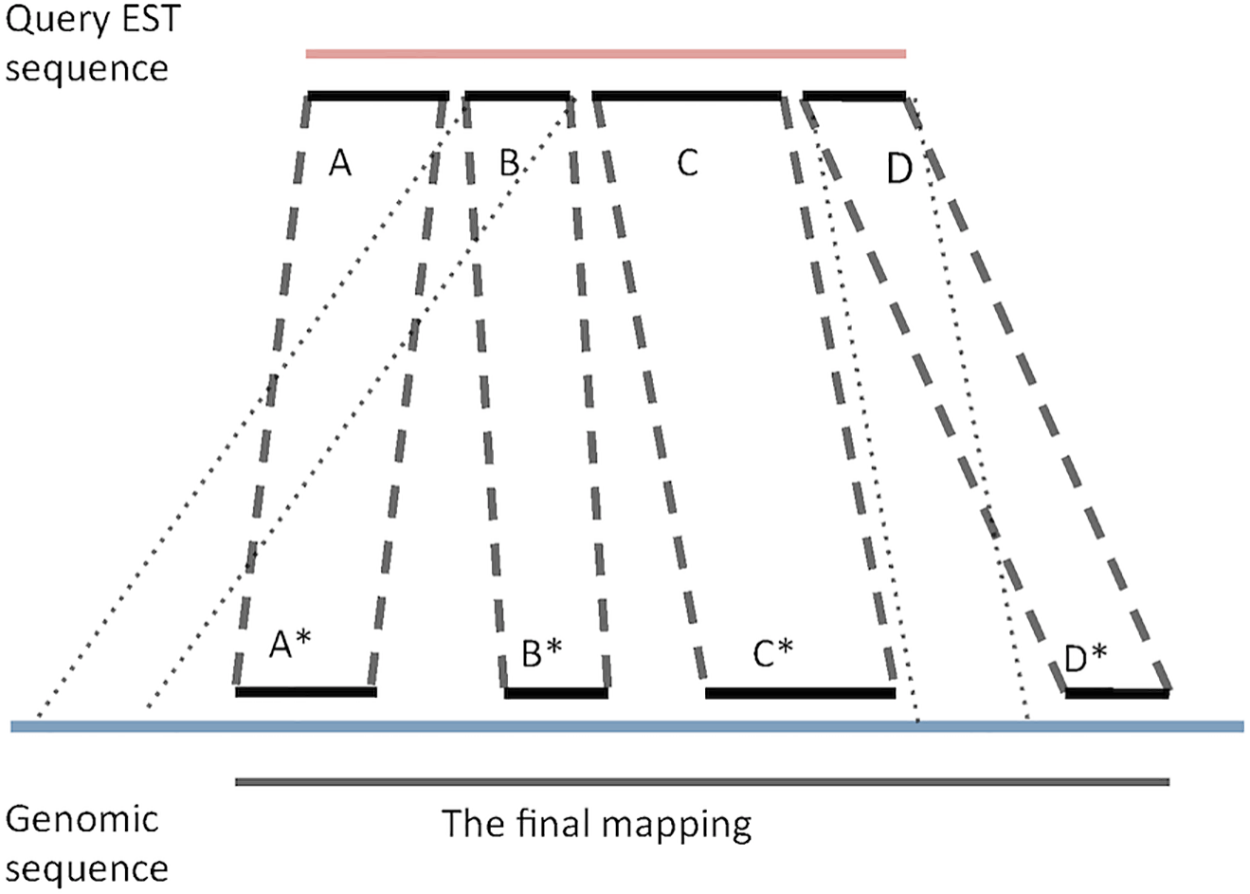
Illustration of EST mapping process. The *HSLA*s (*A*, A*), (*B*, B*), (*C*, C*), and (*D*, D*) are used to report the final mapping.

We study whether soft sampling *k*-mer indexes might increase the speed of these mappers with relatively little loss in sensitivity when working with the human genome as our database. These methods work in two stages. First, they find the set of all HSLAs between an EST and a genome. Then they map the EST to the genome by selecting and linking these HSLAs. The mappers usually differ in how to modify, evaluate, and use the resulting HSLAs to assess the final mapping process. Fig 2 illustrates the mapping procedure.

In this paper, we assess the effectiveness of soft sampling in mapping human ESTs on a human genome. Specifically, we assess whether the correct mapping is retained when we use soft-sampled *k*′-mer indexes to complete the first stage of finding HSLAs. We measure the effect of sampling on both the index size and the query time. We only simulate the mapping process because we want our results to be general and independent from the details of the final mapping process of a mapper. We hope our findings encourage more developers to allow the use of a wider range of *k*′ and *w* values in their mappers.

### Related work

Most previous studies of sampled *k*′-mer indexes have focused on hard sampling with limited study of soft sampling and thus have not studied the effect of choosing a large value of *w* on retention rate, query time, or false positive rate. For example, Morgulis *et al*. [4] built Indexed BLAST, which uses *w* = k* − *k*′ + 1 and supports *k*′ values up to 15. Ning *et al*. [9] built the index SSAHA with *k*′ = 1/2(k* + 1) and *w* = *k*′.

Kent [8] has performed the main previous study of soft sampling. Instead of selecting k* as defined above, Kent developed an analytical model for estimating the likelihood of retaining matches and creating false positives for a variety of indexed search strategies. These include searching with one *k*′-mer, two nearby small *k*′-mers, and one large *k*′-mer with one allowed error. In all cases, he built a soft sampled *k*′-mer index where *w* = k* = *k*′. Kent computed the best choice of *k*′ such that the expected accuracy to find all HSLAs was above a given threshold and the number of shared *k*′-mers that did not lead to HSLAs was as small as possible.

Kent’s work differs from ours in several key ways. First, we consider only *k*′ = 12 so that we can use BLAST to perform our searches, whereas Kent considered multiple *k*′ values. Second, we consider a wide range of *w* values, whereas Kent only considered *w* = *k*′. Thus, Kent’s work does not allow a true study of the effect of *w* on index performance since, in his work, *k*′ is always changing in addition to *w*.

We extend Kent’s analytical model to work with our choices of *k*′ < k* and *w* and we call this new model BLAST model. We compare our empirical accuracy with both Kent’s original model and BLAST model predicted accuracies. Our results show that BLAST model is reasonable accurate in predicting HSLAs retention rate. On the other hand, Kent’s model significantly underestimates the retention rate in our experiments with the human genome. This is expected since Kent’s model is not designed to handle the case when *k*′ < k*.

We focus on fixed sampling where we take every *w*th *k*′-mer occurrence; this is the sampling option supported by NCBI BLAST. An alternative sampling technique is minimizer sampling where we choose a “minimum” *k*′-mer within a given window [21–26]. More specifically, for a window of *w* adjacent *k*′-mers, the *k*′-mer that is alphabetically minimum is selected. The next window then starts one position to right from the previous window.

We focus on fixed sampling for four main reasons. First, it is supported by NCBI BLAST, whereas minimizer sampling is not. Second, constructing a sampled index using fixed sampling is much simpler computationally. Third, fixed sampling reduces the *k*′-mer index size more than minimizer sampling does. Finally, our results indicate that fixed soft sampling achieves good accuracy in finding true matches, so the added complexity of minimizer sampling seems unnecessary when searching for HSLAs.

Our use of *k*′-mers fits within the seed-and-extend searching method. In general, seed-and-extend searching uses two types of seeds: continuous and spaced. We focus on continuous seeds, which are equivalent to *k*-mer seeds. Spaced seeds, seeds that allow mismatches in some predefined positions, increase sensitivity, usually at the cost of greater complexity. We focus on continuous seeds because they minimize the number of false positives without compromising retention rate when searching for HSLAs.

We use *k*′-mer indexes to search for MEM_k_*s. We could use a suffix-tree or a suffix-array index to search for MEM_k_*s instead. But we focus on *k*′-mer indexes because they require less memory than these other indexes, despite the development of many new compressed and sparse suffix array data structures [27, 28]. In particular, a recent paper [29] shows that efficient implementations of a *k*′-mer indexes can find MEM_k_*s more efficiently than compressed sparse suffix arrays, especially when a very large database such as the human genome is used.

Recently, a complementary approach of space-efficient referentially compressed search indexes has been proposed to support similarity searches on genome data sets [11, 12]. In this method, genomes are compressed against some reference genome(s). Given a query, the index then searches two parts: the reference and all genome-specific individual differences. Both parts are saved in compressed suffix trees. Danek *et al*. [13] extend reference-based compression with the use of a *k*′-mer index. We think employing the complementary approach of reference compression in unison with sampled *k*′-mer indexes may be fruitful.

Finally, with respect to mapping ESTs on the human genome, Xin *et al*. [30] proposed two general techniques to accelerate *k*′-mer based mappers. The first technique is to use the set of adjacent *k*′-mers as supporting evidence for the existence of a true match. The second is to use shared infrequent *k*′-mers to select the best mapping location. Similar to other studies, they only used *w* = *k* while evaluating these techniques. In contrast, we test a broader range of *w* values and demonstrate that using a larger *w* greatly reduces query time and index size while suffering only a small loss of sensitivity.

### Problem statement and overall aims

We study the effects of using BLAST with soft sampling when searching for HSLAs and mapping ESTs onto the human genome. Our work is unique in that there is little prior work that has considered soft sampling, and the little work that has considered it has not systematically studied how *w* affects accuracy. We specifically study the effect of sampling parameter *w* on the size, accuracy, and query time of the *k*′-mer index. We also extend previous analytical models to work with our chosen parameters of *k*′ = 12 and *w*, and then compare our empirical results with predictions from both the original and the extended analytical models. We summarize our major contributions.

1. We systematically assess how well BLAST can find *HSLA*(*DB*, *q*, *l*, *t*) when using soft sampling where *w* > k* − *k*′ + 1 when working with the human genome as our database. In particular, we study the retention rates *RR*(*w*) and *RR*_*short*_(*w*). We show that both *RR*(*w*) and *RR*_*short*_(*w*) are high, even for large choices of *w*. Furthermore, the false positive rate, in the form of MEM_k_*s that do not extend into *HSLA*(*DB*, *q*, *l*, *t*), is significantly reduced, leading to a corresponding significant reduction in query time. This demonstrates that soft sampling is a simple but effective method to increase index efficiency with surprisingly little loss in accuracy.
2. We design a new analytical model that we call the BLAST model by extending previously developed analytical models to work with our values of *k*′ < k* and *w*. We compare the theoretical predictions from our new BLAST model and old models with our empirical results. We show that the new model is reasonably accurate whereas other analytical models are not accurate in our context. We also highlight some possible shortcomings of our new BLAST model.
3. Finally, we study the effects of using soft sampling for the problem of mapping human ESTs against the human genome. We conservatively simulate the process because either existing mapping tools do not support soft sampling or do not allow us to replace the first phase aligner. We show that we are able to map more than 98% of the query ESTs perfectly while reducing index size by 3-5 times and query time by 23.3% when compared to hard sampling.

## Materials and methods

We evaluate the effect of soft sampling on using BLAST to (i) find HSLAs and (ii) map ESTs to the human genome. For both applications, we describe our database and how we create sampled indexes. We then describe our query sets and how we perform queries. We next describe our evaluation metrics. Finally, we describe how we extend Kent’s analytical model to work with our choices of *k*′ = 12 and *w*.

### Experimental settings

#### Database

For both applications of finding HSLAs and EST mapping, we use the human genome database provided by Morgulis *et al*. from their MegaBLAST paper [4] as our database. Morgulis *et al*. note that the human genome database was the most frequently searched database in NCBI in 2007 with 10,000 submitted queries per weekday. They partitioned the human genome database into volumes, each volume is roughly 1 GB in size and available at (ftp://ftp.ncbi.nlm.nih.gov/pub/agarwala/indexed_megablast/fasta/human). We summarize key characteristics of each volume in Table 1. We did experiments with both masked and unmasked data but report results only for the unmasked data since the results were similar. As in [4], we treat each volume as a separate database. That is, we create an index for each volume separately and search each volume’s index separately. To obtain results for the human database, we then simply union the results found for each volume.

**Table 1:** Human genome volume characteristics.

Name	Size(Mbytes)	Size(bp)
Chr. 1-5, unmasked	1039.86	1,025,201,451
Chr. 6-13, unmasked	1093.27	1,077,856,590
Chr. 14-Y, unmasked	778.75	767,769,314
Chr. 1-8, masked	1517.93	1,493,033,824
Chr. 9-Y, masked	1400.78	1,377,793,531

ftp://ftp.ncbi.nlm.nih.gov/pub/agarwala/indexed_megablast/fasta/human.

#### Sampled index construction

For finding HSLAs, we use four different minimum alignment lengths *l*: 50, 100, 200, and 400 and a match threshold *t* = 96% or *t* = 97%. For each of our four choices of *l*, we use k* = *l*/(1 + ⌊(1 − *t*)*l*⌋). For mapping ESTs, similar to Kent’s design of BLAT [8], we use the same choices except we omit *l* = 400. Specifically, Kent used *l* = 100; we also include *l* = 50 and *l* = 200 to study EST mapping under a wider set of possible choices. We use a geometric progression with base $\sqrt{2}$ to choose *w* values for soft sampling indexes. Specifically, we consider $w={\sqrt{2}}^{i}$. For each *l*, we ignore *w* less than *w*_0_ = k* − *k*′ + 1 since *w*_0_ is the largest hard sampling value. Likewise, we ignore *w* ≥ *l* as these can completely skip over a potential alignment of length *l*. This results in a total of eleven choices ranging from *w* = 8 to *w* = 256. Combined with four choices of *w*_0_ for hard sampling and three volumes, we create a total of 15 × 3 = 45 sampled indexes.

We use *SI*(*w*) to denote a sampled index created with sampling parameter *w*; note *SI*(*w*_0_) denotes a hard sampling index. These choices are summarized in Table 2. Note some sampled indexes are used with multiple *l* values. For example, the sampled indexes *SI*(22) and *SI*(32) are used for each choice of *l*.

**Table 2:** A summary of the parameters used in our experiments for (1) finding HSLAs and (2) EST mappings. For HSLA, we consider all four choices of *l*. For EST, we only consider the first three choices of *l*.

Sampling parameters					
*l*	*t*	k*	*k*′	*w*_0_	*w* > *w*_0_
50	96%	16	12	5	8, 11, 16, 22, 32
100	97%	25	12	14	16, 22, 32, 45, 64
200	97%	28	12	17	22, 32, 45, 64, 90, 128
400	97%	30	12	19	22, 32, 45, 64, 90, 128, 181, 256

The *k*′-mer indexes are built using BLAST with sampling steps *w*_0_ and *w*. True matches HSLAs are of length ≥ *l* and a match percentage ≥ *t*. Only HSLAs that have shared k*-mers are reported by BLAST.

We build our sampled indexes using the BLAST program makembindex for the three volumes of the unmasked human genome database using BLAST’s default value of *k*′ = 12.

#### Query sets and mappable queries

For HSLA, we use the same query sets that Morgulis *et al*. used to evaluate Indexed BLAST [4]. Morgulis *et al*. organized the queries into three sets based on the average query length: *qsmall* (average length 500), *qmedium* (average length 10,000), and *qlarge* (average length 100,000). Each set has 100 queries for 300 total queries. We group all the queries into a single set of 300 queries and report all results using this single query set. The query sets are available at the following url: ftp://ftp.ncbi.nlm.nih.gov/pub/agarwala/indexed_megablast/queries/human. For EST mapping, we form our query set *Q* by randomly selecting 1000 human ESTs (average length 490) from Expressed Sequence Tags database from NCBI https://www.ncbi.nlm.nih.gov/dbEST. For each length *l*, we define *Q*(*l*) to be the subset of *Q* that has a non-empty *HSLA*(*DB*, *q*, *l*, *t*) and refer to these as the *mappable queries* for length *l*.

#### Query processing

For every query *q* in the query set, we run BLAST using the blastn program with the -task megablast option using its default settings except we select MEM_k_* value using -word-size k*, we use multiple values of *w*, and we set the matching threshold, also known as identity percentage, using -perc-identity 96% for *l* = 50 and -perc-identity 97% for all other *l*. This will return *HSLA*(*DB*, *q*, k*, *t*, *k*′, *w*) where every alignment must have an MEM_k_*. That is, the match percentage *t* will be satisfied, but the lengths are only guaranteed to be at least k*, not *l*. We filter out any HSLAs that are too short to produce *HSLA*(*DB*, *q*, *l*, *t*, *k*′, *w*).

#### False positives

For any query *q* and any *w*, we report the number of false positives *FP*(*q*, *w*) as the number of alignments in *HSLA*(*DB*, *q*, k*, *t*, *k*′, *w*) − *HSLA*(*DB*, *q*, *l*, *t*). This should be very close to the number of MEM_k_*s that do not extend to alignments in *HSLA*(*DB*, *q*, *l*, *t*); the two numbers might differ if multiple MEM_k_*s are part of the same alignment in *HSLA*(*DB*, *q*, k*, *t*) − *HSLA*(*DB*, *q*, *l*, *t*).

#### Experimental system

We run the experiments on a cluster that runs the Community Enterprise Operating System (CentOS) 6.6. The cluster has 24 nodes where each node has two 2.5Ghz 10-core Intel Xeon E5-2670v2 processors and 256 GB memory.

#### HSLA evaluation metrics

We evaluate the effectiveness of a given *k*′-mer index *SI*(*w*) as a function of *w* and *w*_0_ using three metrics: (1) index size reduction, (2) retention rate of HSLAs, and (3) query time reduction. For retention rate, we consider retention of all HSLAs as denoted by *HSLA*(*DB*, *q*, *l*, *t*) and short HSLAs as denoted by *HSLA*_short_(*DB*, *q*, *l*, *t*). To help explain query time reduction, we also measure false positive reduction. We describe each metric in more detail.

For each *SI*(*w*) and each choice of *w* > *w*_0_, we define the sampled index size reduction as

$$\begin{array}{c} SIR\left(w,w_{0}\right)=\frac{\left|SI(w)\right|}{\left|SI(w_{0})\right|} \end{array}$$ (1)

where |*I*| is the size of index *I*. Index size is the sum of dictionary size, measured by counting the number of *k*′-mers, and inverted lists’ size, measured by counting the number of *k*′-mer occurrences in all the inverted lists. Since the human genome is split into three volumes and we create a sampled index for each volume, we compute the total index size for all indexes over all volumes. For the total dictionary size, we take the union of all three dictionaries, and then we measure the total dictionary size by counting the number of *k*′-mers in the union set. For the total inverted lists’ size, we take the sum over all three inverted lists’ sizes.

For each *SI*(*w*) and each choice of *w* > *w*_0_, we report the full retention rate *RR*(*w*, *w*_0_) and the short retention rate *RR*_*short*_(*w*, *w*_0_), which we define as follows. For *w*, *w*_0_ and *q*, we define

$$\begin{array}{c} RR\left(q,w,w_{0}\right)=\frac{\left|HSLA(DB,q,l,t,k^{\prime },w)\right|}{\left|HSLA(DB,q,l,t,k^{\prime },w_{0})\right|} \end{array}$$ (2)

and

$$\begin{array}{c} RR_{short}\left(q,w,w_{0}\right)=\frac{\left|{HSLA}_{\text{short}}(DB,q,l,t,k^{\prime },w)\right|}{\left|{HSLA}_{\text{short}}(DB,q,l,t,k^{\prime },w_{0})\right|} \end{array}$$ (3)

We say that *RR*(*q*, *w*, *w*_0_) or *RR*_*short*_(*q*, *w*, *w*_0_) is undefined if the denominator is 0. We typically report both ratios as percentages. We use all three volumes to get these percentages. We then set *RR*(*w*, *w*_0_) and *RR*_*short*_(*w*, *w*_0_) to be the average of *RR*(*q*, *w*, *w*_0_) and *RR*_*short*_(*q*, *w*, *w*_0_), respectively, where we only include query *q* in the average if *RR*(*q*, *w*, *w*_0_) or *RR*_*short*_(*q*, *w*, *w*_0_), respectively, is defined. We report *RR*(*w*, *w*_0_) since this is a typical user query. We specifically define *RR*_*short*_(*w*, *w*_0_) to fairly compare empirical retention rate with expected retention rate. Intuitively, *RR*_*short*_(*w*, *w*_0_) focuses on the hardest to retain HSLAs.

For each *SI*(*w*) and each choice of *w* > *w*_0_, we report the average query time reduction percentage *QTR*(*w*, *w*_0_), which we define as follows. We start by defining the query time *QT*(*q*, *w*) for a given query *q* and sampled index *SI*(*w*) (including *SI*(*w*_0_)) as follows. We process each query *q* on *SI*(*w*) five times using BLAST and we set *QT*(*q*, *w*) to be the median of the five values. Since *SI*(*w*) is partitioned into three volumes, the query time for a given *q* is the sum of the query times over the three volumes. The query time reduction *QTR*(*q*, *w*, *w*_0_) is then

$$\begin{array}{c} QTR\left(q,w,w_{0}\right)=\frac{QT(q,w)}{QT(q,w_{0})}. \end{array}$$ (4)

Finally, the average query time reduction *QTR*(*w*, *w*_0_) is the average of *QTR*(*q*, *w*, *w*_0_) over all *q*.

Finally, to help explain the query time reduction results, for each *SI*(*w*) and each choice of *w* > *w*_0_, we report the average false positive reduction rate *FPR*(*w*, *w*_0_), which we define as follows. For a given query *q* and sampled index *SI*(*w*) (including *SI*(*w*_0_)), we define *FP*(*q*, *w*) to be the number of false positive; that is, HSLAs that do not lead to elements of *HSLA*(*DB*, *q*, *l*, *t*) when we apply the second, more expensive, extension phase. We believe that *FP*(*q*, *w*) decreases as *w* increases, and this may help explain any reduction in query time. To test this, we define the false positive reduction rate *FPR*(*q*, *w*, *w*_0_) to be

$$\begin{array}{c} FPR\left(q,w,w_{0}\right)=\frac{FP(q,w)}{FP(q,w_{0})} \end{array}$$ (5)

Finally, the average false positive reduction rate *FPR*(*w*, *w*_0_) is the average of *FPR*(*q*, *w*) over all *q*.

#### EST mapping evaluation metrics

For each soft sampled index *SI*(*w*) and a given length *l*, we report its retention rate, *RR*_*map*_(*w*, *l*), as the percentage of *Q*(*l*) such that *all* of *HSLA*(*DB*, *q*, *l*, *t*) is found using *SI*(*w*). We use this requirement because this implies that the mapping result for *SI*(*w*) for the given query *q* will be identical to the mapping result for *SI*(*w*_0_) and *q* regardless of the mapping procedure used. Otherwise, at least one highly similar local alignment is lost and we pessimistically assume that the mapping result would be lost as well.

More formally, for a given mappable queries set *Q*(*l*) and *k*′-mer index *SI*(*w*), we define the set *Q*′(*l*) ⊂ *Q*(*l*) as follows

$$\begin{array}{c} Q^{\prime }\left(l\right)=\left\{q\in Q\left(l\right)|HSLA\left(DB,q,l,t,k^{\prime },w\right)=HSLA\left(DB,q,l,t,k^{\prime },w_{0}\right)\right\} \end{array}$$ (6)

Then, we define the index retention rate *RR*_*map*_ as follows:

$$\begin{array}{c} RR_{map}\left(w,w_{0}\right)=\frac{|Q^{\prime }\left(l\right)|}{\left|Q(l)\right|} \end{array}$$ (7)

We also report the effect of *w* on query time using the same process as with HSLA, namely, running each query five times, taking the median time, and then reporting the average reduction in query time over all 1000 queries. Note that we use all queries rather than just the mappable queries when reporting query time.

### Analytical modeling

We now describe how we analytically model two of the evaluation metrics, index size reduction and retention rate.

#### Predicting index size

We first show how we compute the expected size of a sampled index *SI*(*w*). For the dictionary, we assume the *k*′-mer dictionary is full and thus the size of a *k*′-mer dictionary is 4^*k*′^ entries, which in our case, is 4^12^. This may not be accurate, but since the dictionary size is typically much smaller than the inverted lists size given *k*′ = 12, this is accurate enough. The number of *k*′-mer occurrences stored in the inverted lists is simply (*D* − *S*(*k*′ + 1))/*w* where *D* = |*DB*|, the number of positions in *DB*, and *S* is the number of distinct sequences in *DB*. Thus, the predicted size of *SI*(*w*) is simply

$$\begin{array}{c} size\left(SI\left(w\right)\right)=4^{k^{\prime }}+\frac{D-S(k^{\prime }+1)}{w} \end{array}$$ (8)

#### Predicting retention rate

We present a new analytical model to compute the expected retention rate of HSLAs in *HSLA*_short_(*DB*, *q*, *l*, *t*). We start by presenting Kent’s analytical model [8] where he essentially assumed *w* = *k*′ = k* in his model. We then propose a new model that we refer to as the BLAST model to account for typical parameters used in BLAST searches. We refer to the expected retention rates as *E*[*RR*_*K*_] and *E*[*RR*_*B*_] for Kent’s model and our model, respectively. For both retention rates, we make a few simplifying assumptions and refer to the two models generically as *E*[*RR*] when describing these common assumptions. First, we restrict our attention to HSLAs that have length exactly *l*. Second, we assume each HSLA in *HSLA*_short_(*DB*, *q*, *l*, *t*, *k*′, *w*) is retained with the same probability, and this probability is independent of other HSLAs. This implies

$$\begin{array}{c} E\left[RR\right]=E\left[\frac{\left|{HSLA}_{\text{short}}(DB,q,l,t,k^{\prime },w)\right|}{\left|{HSLA}_{\text{short}}(DB,q,l,t)\right|}\right] \end{array}$$ (9)

which simplifies to just *p*(*A*)

$$\begin{array}{c} E[RR]=p(A) \end{array}$$ (10)

The *p*(*A*) represents the probability that a short HSLA *A* is retained. This allows us to focus on a single short HSLA *A* in the rest of this analysis. Finally, we assume each position in *A* is independent of other positions and the probability that any position in *A* is a match is exactly *t*.

#### Kent’s original retention rate model (*E*[*RR*_*K*_])

We start with Kent’s original model [8]. where he assumes *w* = k* = *k*′. The number of k*-mers that are guaranteed to be chosen from *x* within *A* is

$$\begin{array}{c} T=\left\lfloor (|x\right|-k^{*}+1)/w\rfloor \end{array}$$ (11)

Furthermore, these k*-mers will be adjacent to each other with no gaps. For *A* to be retained, at least one of these chosen k*-mers from *x* must exactly match the corresponding k*-mer in *y* from *q*. The probability of such an exact match assuming each position is independent and that the overall match percentage within *A* is *t* is then

$$\begin{array}{c} p=t^{k^{*}} \end{array}$$ (12)

Since the sampled k*-mers do not overlap, the probability that all fail to match is then (1 − *p*)^*T*^. Thus, the probability that at least one will match and alignment *A* will be found is *p*(*A*) = 1 − (1 − *p*)^*T*^. Since *E*[*RR*_*K*_] = *p*(*A*), we have

$$\begin{array}{c} E\left[RR_{K}\right]=1-{\left(1-p\right)}^{T} \end{array}$$ (13)

#### BLAST retention rate model (*E*[*RR*_*B*_])

To extend this analysis to the typical BLAST setting with distinct *w*, k* and *k*′, we must modify the formula in two ways. The first key issue is that we sample *k*′-mers but then extend them to search for k*-mers. The sampled *k*′-mer must be an exact match, which again happens with probability *p* = *t*^*k′*^ The key issue after this is whether this can be extended to an MEM_k_*. Suppose this can extend exactly 0 ≤ *l* ≤ k* − *k*′ − 1 characters to the left before we get a mismatch. We then need it to extend at least k* − *l* − *k*′ characters to the right. The probability we can extend exactly *l* characters to the left is *t*^*l*^(1 − *t*). The probability we can extend at least k* − *l* − *k*′ characters to the right is *t*^k*^^−*k*′^^−*l*^. Thus, the probability that we have a *k*′-mer, it extends exactly 0 ≤ *l* ≤ k* − *k*′ − 1 characters to the left, and it extends at least k* − *l* − *k*′ characters to the right is then *t*^*k*′^*t*^*l*^(1 − *t*)*t*^k*^^−*k*′^^−*l*^ = *t*^k*^(1 − *t*) There are k* − *k*′ − 1 choices for *l* leading to a final probability of (k* − *k*′ + 1)*t*^k*^(1 − *t*). The other possibility is that it extends at least k* − *k*′ characters to the left, which occurs with probability *t*^k*^ giving us a total probability of

$$\begin{array}{c} p^{\prime }=\left(k^{*}-k^{\prime }-1\right)t^{k^{*}}\left(1-t\right)+t^{k^{*}} \end{array}$$ (14)

The second key issue is that in Eq 11, we used the floor function as this is the number of k*-mers from *x* within *A* that are guaranteed to be chosen. Using the floor function ignores the possibility that we may have an additional k*-mer chosen from *x*. That is, the number of k*-mers from *k* that will be sampled might be either

$$\begin{array}{c} T_{f}=\left\lfloor (|x\right|-k^{*}+1)/w\rfloor \end{array}$$ (15)

$$\begin{array}{c} T_{c}=\left\lceil (|x\right|-k^{*}+1)/w\rceil \end{array}$$ (16)

where *T*_*f*_ = *T* from Eq 11. If we assume that each possible window for *w* is equally likely, then

$$\begin{array}{c} p\left(T_{f}\right)=\frac{w\times T_{c}-\left(|x\right|-k^{*}+1)}{w} \end{array}$$ (17)

$$\begin{array}{c} p\left(T_{c}\right)=1-p\left(T_{f}\right) \end{array}$$ (18)

For the case where ⌊(|*x*| − k* + 1)/*w*⌋ = (|*x*| − k* + 1)/*w*, *p*(*T*_*f*_) = 0, which means *p*(*T*_*c*_) = 1 so the result is still correct.

In our new BLAST model, we update Eq 13 replacing *p* with *p*′ and replacing *T* with *T*_*c*_ and *T*_*f*_ as follows.

$$\begin{array}{c} E\left[RR_{B}\right]=1-p\left(T_{f}\right){\left(1-p^{\prime }\right)}^{T_{f}}-p\left(T_{c}\right){\left(1-p^{\prime }\right)}^{T_{c}} \end{array}$$ (19)

We will compare both Kent’s model and our new BLAST model in our results.

## Results and discussion

We report the impact of sampling on the efficiency of a *k*′-mer index on the index size and query performance. We report both the expected and the actual impact of sampling.

### Index size

*As expected, the index size is inversely proportional to the sampling step* *w*. This means that soft sampling does lead to a significant reduction in space when compared to hard sampling. For example, when *w*/*w*_0_ is roughly 1.7 and 4.4, the index size reduces by 38% and 74% for all values of *l* we considered. The percentage of reduction increases as *l* increases. For example when *l* = 400 and *w*/*w*_0_ is almost 10, the index size reduces by 90%.

**Fig 3:**
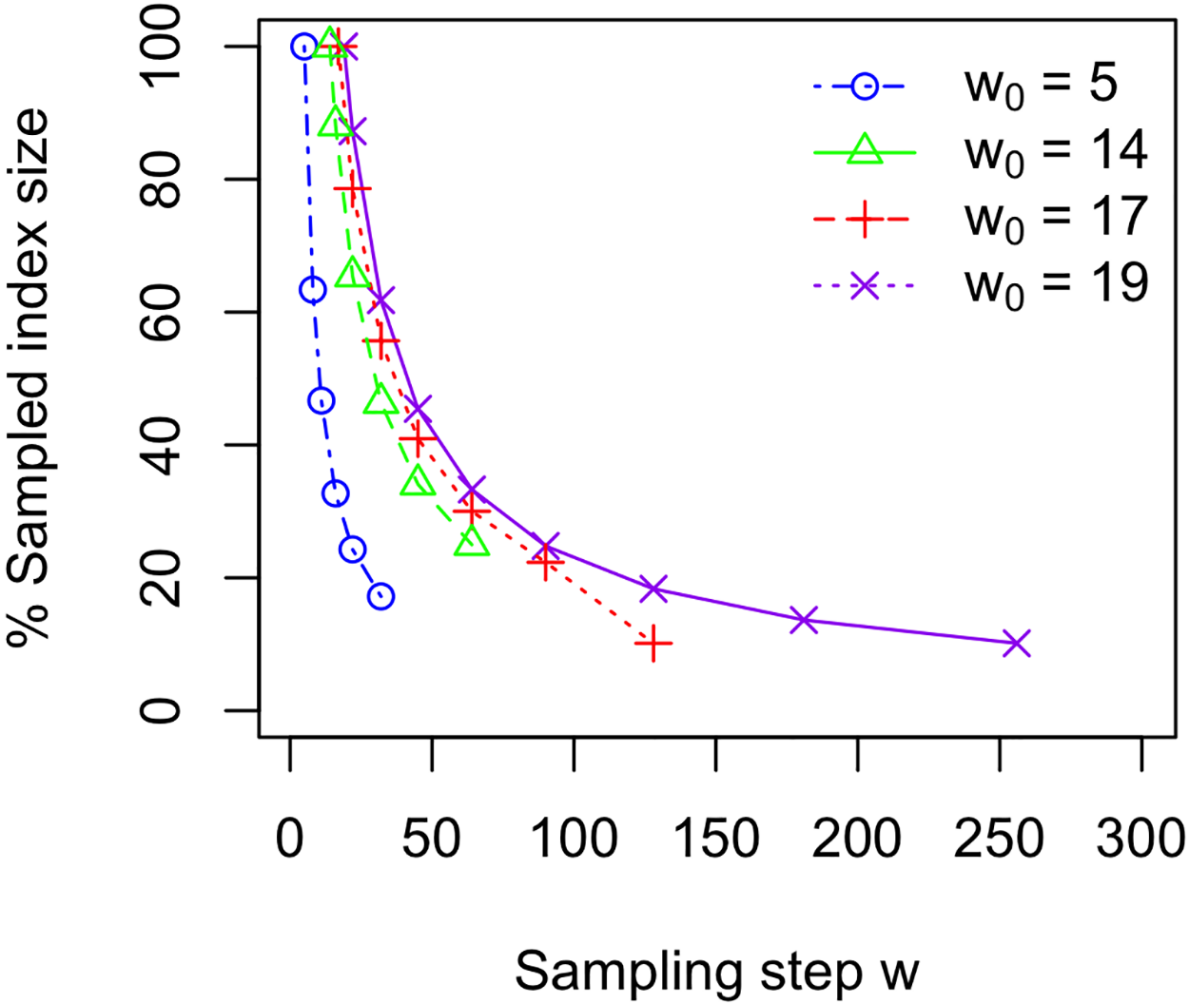
The sampled index *SI*(*w*) size (percentage) as a function of sampling step size *w* of *SI*(*w*) versus sampled index *SI*(*w*_0_). The *k*′-mer indexes are built with *k*′ = 12 and *w* ≥ *w*_0_ where *w*_0_ = *l* − *k* + 1.

With hard sampling *w*_0_ = k* − *k*′ + 1, the space reduction is limited by k*. With soft sampling *w* > k* − *k*′ + 1, *w* is limited primarily by *l*, where typically *l* ≫ k* (see Table 2). We plot results for the percentage reduction in index size in Fig 3. Since the expected index size (see Eq 8) and the actual index size are almost identical, the expected size is omitted.

Sampling reduces index size because it reduces the number of sampled *k*′-mers leading to a factor of *w* reduction in inverted lists size, the dominant component of index size. On the other hand, although sampling does reduce dictionary size, the reduction is relatively small and does not greatly affect the final index size. For example when *w*/*w*_0_ is roughly 1.7 and 4.4, the dictionary is about 9% and 17% of the the index size and the average reduction in the dictionary size is 9% and 27% respectively.

### Retention rate of *HSLA*s

**Fig 4:**
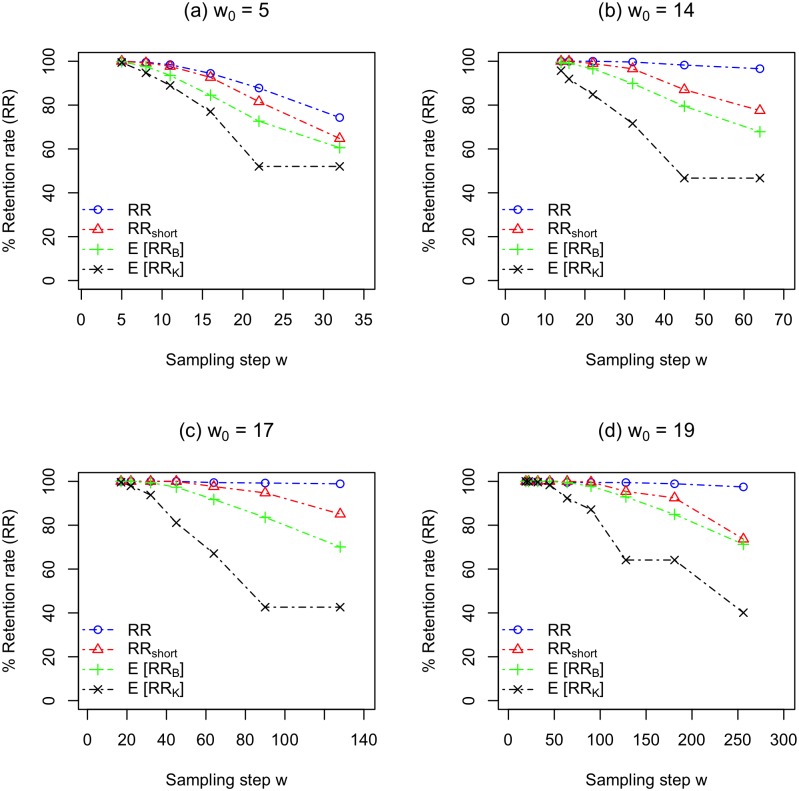
The actual HSLA retention rate *RR*(*w*, *w*_0_), the actual short HSLA retention rate *RR*_*short*_(*w*, *w*_0_), and the expected short HSLA retention rate using both Kent’s model *E*[*RR*_*K*_(*w*, *w*_0_)] and BLAST model *E*[*RR*_*B*_(*w*, *w*_0_)] for (a) *l* = 50, *w*_0_ = 5, (b) *l* = 100, *w*_0_ = 14, (c) *l* = 200, *w*_0_ = 17, and (d) *l* = 400, *w*_0_ = 30. For other parameters values see Table 2.

We first examine *RR*(*w*, *w*_0_) to study how the overall HSLA retention rate changes as a function of *w*, *w*_0_, and *l*. We first observe that *RR*(*w*, *w*_0_) improves as *l* increases. In particular, as can be seen from our *RR*(*w*, *w*_0_) results from Fig 4, if we look at choices for *w* and *w*_0_ that have a similar ratio *w*/*w*_0_, the *RR*(*w*, *w*_0_) retention result is higher for larger *l*. In particular, whereas *RR*(32, 5) for *l* = 50 falls below 80%, *RR*(*w*, *w*_0_) ≥ 96.6% for *l* ≥ 100 for all tested values of *w*, and *RR*(256, 30) = 97.5% for *l* = 400. Thus, for large values of *l*, we can use soft sampling where *w*/*w*_0_ approaches even 10 and still achieve retention rates of close to 100%.

We now focus on the retention rate of short HSLAs. First we compare *RR*(*w*, *w*_0_) with *RR*_*short*_(*w*, *w*_0_) as a function of *w*, *w*_0_, and *l*. Fig 4 also contains our *RR*_*short*_(*w*, *w*_0_) results. *We observe that if* *w*/*w*0 < 4, *then the difference between* *RR*(*w*, *w*_0_) *and* *RR*_*short*_(*w*, *w*_0_) *is small (less than 4%) for almost all choices of* *l*; the one outlier is *l* = 100 where we see a difference of 11% for *w*/*w*_0_ = 45/14 ≈ 3.2. For example, for all values of *l* except 100, the difference between *RR*(*w*, *w*_0_) and *RR*_*short*_(*w*, *w*_0_) is at most 1.8%. We do see a significant difference between *RR*(*w*, *w*_0_) and *RR*_*short*_(*w*, *w*_0_) for our largest choices of *w*, which means that *RR*_*short*_(*w*, *w*_0_) does fall off by *w*/*w*_0_ = 10 or so. Thus, in contrast to *RR*(*w*, *w*_0_), there is an upper limit to how much we can soft sample before *RR*_*short*_(*w*, *w*_0_) suffers.

Next we examine how *RR*_*short*_(*w*, *w*_0_) changes as a function of *w*, *w*_0_, and *l*. Similar to *RR*(*w*, *w*_0_), we observe *RR*_*short*_(*w*, *w*_0_) generally improves as *l* increases given roughly the same ratio of *w*/*w*_0_. For example, when *w*/*w*_0_ is roughly 6, *RR*_*short*_(*w*, *w*_0_) is 65%, and 85%, and 95% for *l* equal to 50, 200, and 400, respectively. In general, we can retain 90% more short HSLAs for either small *w*/*w*_0_ ratios (less than 2 or 3) or large *l* values (200 or 400).

We now want to compare empirical retention rate with predicted retention rate as a function of *w*, *w*_0_ and *l*. Comparing *RR*(*w*, *w*_0_) to *E*[*RR*_*B*_(*w*, *w*_0_)] is not fair as *RR*(*w*, *w*_0_) includes many alignments significantly longer than *l* whereas *E*[*RR*_*B*_(*w*, *w*_0_)] focuses only on alignments with length exactly *l*. To more fairly compare empirical retention rate to expected retention rate, we compare *RR*_*short*_(*w*, *w*_0_), where the length of the alignment is in the range [*l*, (2 − *t*)*l*], with *E*[*RR*_*B*_(*w*, *w*_0_)], where an alignment is assumed to have a length *l*. We consider HSLAs with length up to (2 − *t*)*l* to ensure there are a reasonable number of HSLAs. We also note that if we assume that the (1 − *t*)*l* errors were all insertions rather than substitutions, this would increase the length of the HSLA to (2 − *t*)*l*. Fig 4 also contains our *E*[*RR*_*B*_(*w*, *w*_0_)] results.

*We observe that the BLAST model predicts actual retention rate of short HSLAs with reasonable accuracy*, *particularly for small* *w*/*w*_0_ *and for larger* *l*. For example, the typical difference between *RR*_*short*_(*w*, *w*_0_) and *E*[*RR*_*B*_(*w*, *w*_0_)] when *w*/*w*_0_ ≤ 2 is less than 5% for all choices of *l* and is less than 1% for large *l* = 200 and *l* = 400. The typical difference stays below 10% for almost all choices of *w*/*w*_0_ and *l* with only a few exceptions. The difference between *RR*_*short*_(*w*, *w*_0_) and *E*[*RR*_*B*_(*w*, *w*_0_)] does grow as *w*/*w*_0_ increases, but at a relatively slow rate, typically maximized at the largest choice of *w*/*w*_0_, though this does not hold for *l* = 400. We do note that we have relatively few empirical HSLAs for the large *w* values for *l* = 400, so perhaps with more samples, the difference between *RR*_*short*_(*w*, *w*_0_) and *E*[*RR*_*B*_(*w*, *w*_0_)] might increase for these *l* and *w* choices.

Finally, we compare the predictions from Kent’s model *E*[*RR*_*K*_(*w*, *w*_0_)] and our new BLAST model *E*[*RR*_*B*_(*w*, *w*_0_)]. *Our new BLAST model is significantly more accurate than Kent’s model, especially as* *w*/*w*_0_ *increases*. For example, for *w*/*w*_0_ equal to 1.7, 3.4 and 4.7, *E*[*RR*_*K*_] is on average less than *E*[*RR*_*B*_] by 5%, 18%, and 23%, respectively, for all *l* values we consider. Kent’s model has several issues. First, because of the floor function used in Eq 11, it underestimates the number of sampled k*-mers from a given HSLA resulting in common retention rate predictions for multiple values of *w*. For example when *l* = 100, *E*[*RR*_*K*_] = 46.70% for both *w* = 45 and *w* = 64. The second flaw is that Kent’s model was not designed to handle different values for *k*′, k*, and *w*, which is what is typically used in BLAST. Because our BLAST model is designed to overcome both issues, it achieves better results, particularly for larger *w*/*w*_0_ and for larger *l*.

### Possible improvements for the BLAST model

While our new BLAST model is much more accurate than Kent’s original model, it still underestimates actual retention rates for large *w*/*w*_0_. We now explore possible explanations for this underestimate. We believe the fundamental problem with our new BLAST model (as well as Kent’s model) is that for any *HSLA*_short_(*DB*, *q*, *l*, *t*), it only assumes that each position is a match with probability *t*.

We demonstrate the shortcomings of this assumption in two different ways. We first show that using this assumption, we greatly underestimate the length of the maximum MEM within any HSLA; we refer to this maximum MEM length as MAX-MEM. Long MEMs are relevant because long MEMs significantly increase the likelihood of recovering an HSLA. For example, if an HSLA includes an MEM of length *w* + *k* − 1, then it is guaranteed the HSLA will be found since one *k*-mer is guaranteed to be chosen from within the MEM.

We perform this comparison as follows. We first obtain an empirical distribution of MAX-MEM by recording the length of the longest MEM in every HSLA in *RR*_*short*_(*w*, *w*_0_). We then use the BLAST model’s fundamental assumption that each position in an HSLA is identical with probability *t* to create a corresponding predicted distribution of MAX-MEM. For this predicted distribution of MAX-MEM, we assume that the length of the HSLA is *l*, the number of mismatches is exactly (1 − *t*)*l*, and each position is equally likely to be a mismatch. All told, there are *l* choose (1 − *t*)*l* different combinations of errors that are equally likely. We can then compute the predicted distribution by enumerating all possibilities for *l* = 50 and *l* = 100.

For *l* > 100, it takes too much time to enumerate all possibilities. Thus, we use Monte Carlo simulation to compute a second predicted distribution for MAX-MEM. We create short HSLAs as follows. We start with an alignment of length *l*, a set *S* of mismatch positions, which is initialized to empty, and a count *C* of the number of mismatch positions, which is initially 0. Then, we repeatedly choose a position in the range [0, *L* − 1] uniformly at random. If the position is not in *S*, we add the position to *S* and increment *C* by one. Otherwise, we do nothing with the chosen position and choose another one. When *C* reaches (1 − *t*)*l*, we stop with a complete HSLA. We then record its longest MEM. We do this until we have recorded one million such longest MEMs.

**Fig 5:**
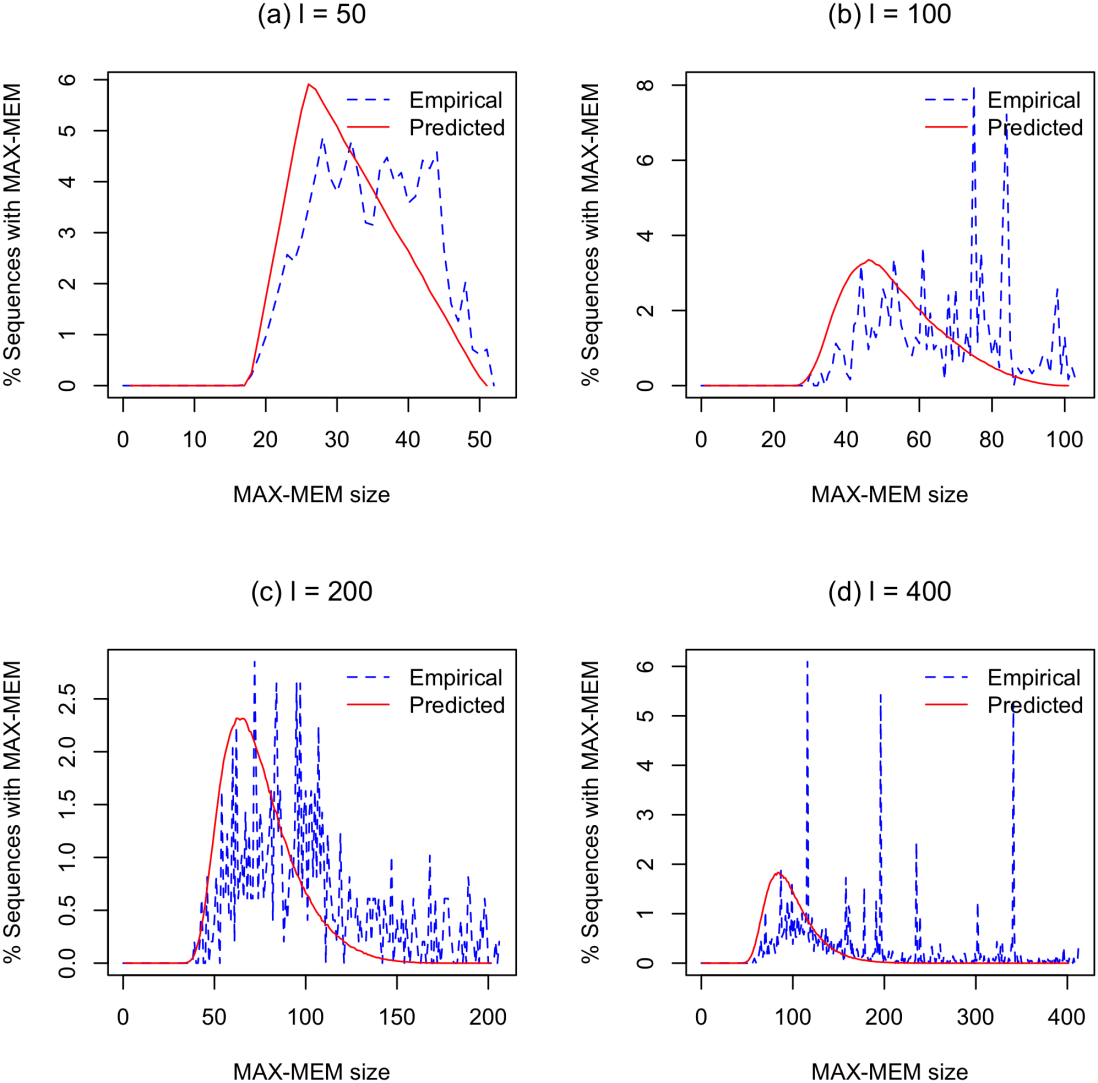
Distribution of predicted and empirical MAX-MEM lengths in HSLAs. The predicted MAX-MEM lengths are computed from a Monte Carlo simulation. (a) *l* = 50, *t* = 96%, (b) *l* = 100, *t* = 97%, (c) *l* = 200, *t* = 97%, and (d) *l* = 400, *t* = 97%.

For *l* = 50 and *l* = 100, Monte Carlo simulation and complete enumeration produce essentially identical distributions for MAX-MEM. Thus, we only show results from our Monte Carlo simulations since these cover all choices of *l*. We show the results for our experimental and Monte Carlo distribution of MAX-MEM for all four choices of *l* in Fig 5. We observe that the empirical MAX-MEM distribution is weighted more heavily towards longer MEMs than the predicted distribution. This demonstrates that the assumptions used in the BLAST model do not correctly predict the distribution for MAX-MEM length; in general, they underestimate the probability for finding longer MEMs. While the distribution of MAX-MEM is not identical to retention rate of HSLAs, this finding provides evidence that we need stronger assumptions to better predict retention rate of HSLAs.

We now show another fundamental flaw with the base assumption of the BLAST model. Consider an HSLA of length *l*, and suppose we assume that each query position is independent and matches its corresponding database position with probability *t* (similar to the BLAST model’s assumptions). Then, there is a (1 − *t*) probability that each position does not match. In this scenario, the total number of mismatches has the binomial distribution *Bin*(*l*, (1 − *t*)), which has an expected number of mismatches of exactly (1 − *t*)*l*. If the number of mismatches exceeds this expected value, we would no longer have an HSLA, but this is clearly contradicts with the first assumption that we start with an HSLA. The probability that the number of mismatches exceeds (1 − *t*)*l* is given in Table 3. Given this weak assumption, the BLAST model essentially starts with a probability ranging from 32% to 43% that the given HSLA is not an HSLA.

**Table 3:** The probability that the number of mismatches exceeds (1 − *t*)*l* for various choices of *l* and *t*.

*l*	*t*	Probablity mismatches exceeds (1 − *t*)*l*
50	0.96	32.3%
100	0.97	35.3%
200	0.97	39.4%
400	0.97	42.4%

Under the assumption that the number of mismatches in a HSLA follow *Bin*(*l*, (1 − *t*)), Kents’s model underestimates the the existence of the HSLA by 30%–40%.

We have shown that the weak assumption used in the BLAST model (1)underestimates the probability of longer MAX-MEMs and (2) gives a significant probability for HSLAs to not be HSLAs. Taken together, we believe a new model with stronger assumptions is needed to produce more accurate predictions about retention rate of short HSLAs.

### Query time

**Fig 6:**
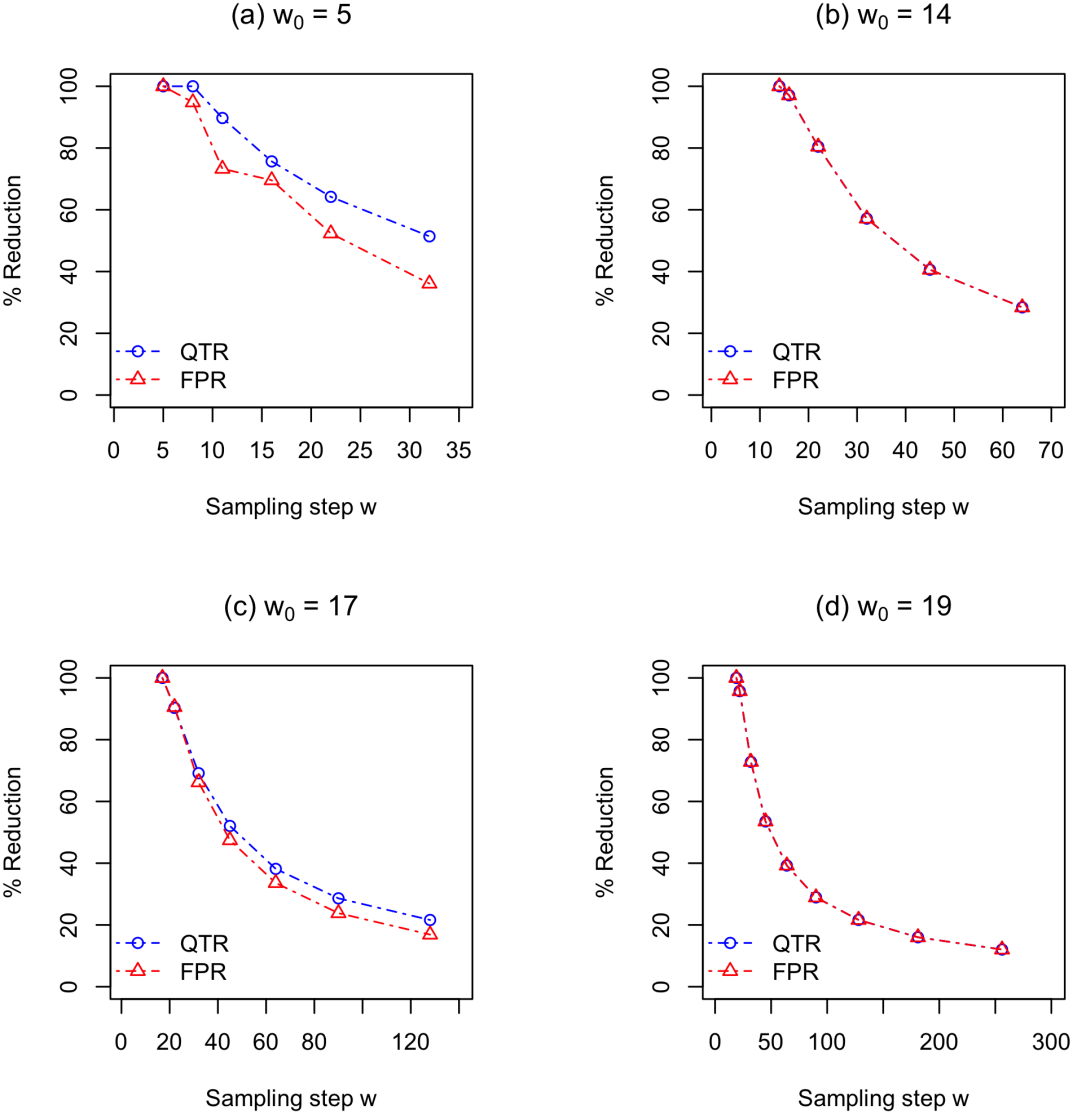
The average median query time reduction (QTR) percentages and the actual false positive reduction (FPR) percentages as a function of sampling step *w*. (a) *l* = 50, *w*_0_ = 5, (b) *l* = 100, *w*_0_ = 14, (c) *l* = 200, *w*_0_ = 17, and (d) *l* = 400, *w*_0_ = 30. For other parameters values see Table 2.

We now examine how soft sampling affects query time. *The query time is approximately inversely proportional to the sampling step w for all l values without significantly reducing retention rate RR*(*w*, *w*_0_). For example, for *l* = 200, the median query time for hard sampling is 26 hours. When *w*/*w*_0_ is 3.8 and 5.3, the median query time reduction percentages are 64.51% (median query time 10 hours) and 73.38% (median query time 7.4 hours), respectively, while maintaining *RR* > 99%. Similarly, for *l* = 400, the median query time for hard sampling is 18.6 hours. When *w*/*w*_0_ is 6.7 and 9.3, the query time reduction percentages are 78.36% (median query time 4.3 hours) and 83.99% (median query time 3.3 hours), respectively, while maintaining *RR* > 99%. Fig 6 shows our full query time results represented as query time reduction (*QTR*) percentages.

The reduction in false positive rate mostly explains the reduction in query time. Recall that false positives are alignments in *HSLA*(*DB*, *q*, k*, *t*) − *HSLA*(*DB*, *q*, *l*, *t*), which is roughly the number of MEM_k_*s that do not extend into alignments in *HSLA*(*DB*, *q*, *l*, *t*). This implies much of the query time is spent ruling out false positives and that using soft sampling not only has little affect on retention rate but also significantly reduces false positives. Our full false positive reduction rate results are shown in Fig 6. As can be seen from this figure, the plots for false positive reduction rate (*FPR*) and query time reduction (*QTR*) percentage are very similar.

### Mapping results

We report our retention rate and query time results for EST mapping in Tables 4, 5 and 6. *Our results show that the number of mappable queries that retain all HSLAs is very high even when we use soft sampling*. Furthermore, we achieve significant reductions in query processing time. Recall that a query *q* is mappable is there is at least one HSLA between *q* and the reference (the human genome in our case). When an index *SI*(*w*) is used where *w* > *w*_0_, a query is lost if even a single HSLA is not found by *SI*(*w*).

**Table 4:** The retention rate (*RR*_*map*_) and query time reduction (*QTR*) results for all 1000 queries when *l* = 50 and *w*_0_ = 5, where 879 were mappable queries.

*w*	# retained queries	*RR*_*map*_	*QTR*
5	879	100.00%	100.00%
8	876	99.66%	100.00%
11	864	98.29%	89.72%
16	849	96.59%	75.67%
22	811	92.26%	64.19%
32	677	77.02%	51.40%

**Table 5:** The retention rate (*RR*_*map*_)and query time reduction (*QTR*) results for all 1000 queries when *l* = 100 and *w*_0_ = 14, where 794 were mappable queries.

*w*	# retained queries	*RR*_*map*_	*QTR*
14	794	100.00%	100.00%
16	794	100.00%	95.60%
22	794	100.00%	85.67%
32	793	99.87%	83.07%
45	789	99.37%	80.26%
64	784	98.74%	76.68%

**Table 6:** The retention rate (*RR*_*map*_)and query time reduction (*QTR*) results for all 1000 queries when *l* = 200 and and *w*_0_ = 17, where 528 were mappable queries.

*w*	# retained queries	*RR*_*map*_	*QTR*
17	528	100.00%	100.00%
22	528	100.00%	93.76%
32	528	100.00%	91.91%
45	525	99.43%	87.07%
64	528	100.00%	84.08%
90	523	99.05%	80.36%
128	506	95.83%	78.76%

Using soft sampling, we are able to greatly reduce the index size, significantly reduce query time, and correctly map more than 95% of the mappable queries for *l* ≥ 100. In fact, for *l* ≥ 100, we correctly map almost 99% of the mappable queries for *w*/*w*_0_ approaching 5. For *l* = 200, we correctly map at least 95% of the mappable queries for *w*/*w*_0_ = 7.5. For *l* = 50, we still see good retention rates but the drop off is a bit faster. Specifically, for *l* = 50, for *w*/*w*_0_ approaching 3, we correctly map 96% of the mappable queries. For *w*/*w*_0_ between 4 and 5, we correctly map 92% of the mappable queries, and for *w*/*w*_0_ between 6 and 7, we correctly map 77% of the mappable queries. Finally, the actual retention rates may be even better than the ones reported as we required that all HSLAs be retained whereas mapping may proceed properly even if some HSLAs are lost.

In human EST mapping, it is common to use HSLAs of length *l* = 100 to search for the best mapping [8–10]. To study a broader range of possibilities, we also consider *l* = 50 (more HSLAs and thus more mappable queries) and *l* = 200 (fewer HSLAs and thus fewer mappable queries). Our results imply that soft sampling can be used with relatively small loss in sensitivity for the commonly used case of *l* = 100. Given that the index sizes are significantly reduced and query times are also reduced, soft sampling may allow for EST mapping using *k*-mer based methods for larger genomes with only a small loss in sensitivity.

## Conclusion

We now summarize our main conclusions. Based on our experiments with the human genome and NCBI BLAST, soft sampling achieves significant space and time savings while also retaining highly similar local alignments with much higher probabilities than predicted by analytical modeling. Even better, when applied to EST mapping, soft sampling achieves significant space and time savings while retaining 98% of all mapping results (for *l* = 100), and the retention results may be even better as we pessimistically assume that losing even a single highly similar local alignment will lead to an incorrect mapping result. Further, because we performed all of our local alignments using BLAST, these results can be easily tested and adopted by other researchers.

This is but a first step in studying soft sampling. We can extend this work in many directions. We would like to test the effectiveness of soft sampling using other real biological data sets and in other applications such as clustering or SNP detection. Also, we would like to combine soft sampling with reference-based compressed *k*-mer indexes, which are useful when there is high redundancy in the data sets [11–13]. We focused on soft fixed sampling. An alternative method is soft minimizer sampling, which uses lexicographic information during the sampling process and allows sampling of query *k*-mers. In future research we plan to compare the two methods.
